# Computer vision syndrome and predictors among computer users in Ethiopia: a systematic review and meta-analysis

**DOI:** 10.1186/s41182-022-00418-3

**Published:** 2022-03-24

**Authors:** Fentahun Adane, Yoseph Merkeb Alamneh, Melaku Desta

**Affiliations:** 1 Department of Biomedical Sciences, School of Medicine, Debre Markos University, Debre Markos, Ethiopia; 2 Department of Midwifery, College of Health Science, Debre Markos University, Debre Markos, Ethiopia

**Keywords:** Computer vision syndrome, CVS, Secretaries, Bankers, Employees, System review, Meta-analysis and Ethiopia

## Abstract

**Background:**

A computer is one of the most widely used office tools. The leading occupational health problem of the twenty-first century is computer vision syndrome (CVS). Research findings across Ethiopia on the magnitude and predictors of CVS among computer users are highly variable and inconsistent. Therefore, this study aimed to estimate the overall prevalence of CVS and its predictors among computer users in Ethiopia.

**Methods:**

We searched articles in all databases and other sources. Cochrane Q test statistics and I^2^ tests were used. A random-effect meta-analysis model was used. In addition, the association between risk factors and CVS among computer users was examined.

**Results:**

Eight eligible studies were included. The pooled prevalence of CVS among computer users in Ethiopia was 73.21% (95% CI 70.32–76.11). Sub-group analysis by profession has shown that the highest prevalence of CVS was observed in bank employees [73.76% (95% CI 70.40–77.13)]. The most common reported symptoms of CVS were blurred vision (34.26%; 95% CI 22.08, 46.43). The previous history of eye disease (95% CI 2.30, 5.47), inappropriate sitting position (95% CI 1.76, 3.22), the frequent use of a computer (95% CI 2.04, 3.60), and using eyeglass/spectacles (95% CI 1.10, 3.91) were significantly associated with CVS among computer users in Ethiopia.

**Conclusions:**

According to this study, computer vision syndrome was high among computer users in Ethiopia. Computer vision syndrome (CVS) was significantly associated with a previous history of eye disease, inappropriate sitting position, frequent use of a computer, and the use of spectacles. Based on the findings, it is suggested that efforts be made to optimize computer exposure time. It is also worth noting that employees should be properly seated when using a computer. Furthermore, people with vision problems should be extra cautious when using a computer. Finally, community awareness of the safety precautions that can be taken to reduce CVS is critical.

## Background

Computers were one of the most widely used workplace tools in the world. In the twenty-first century, it had become a requirement, and it was routinely utilized in a variety of organizations, including academic institutions, government offices, and the financial system [1].

Computer vision syndrome(CVS) is caused by prolonged usage of a computer [2]. The American Optometric Association (AOA) states computer vision syndrome (CVS) as a collection of eye and vision disorders caused by activities that strain near vision and that occur in conjunction with or during the use of computers [3]. It refers to a set of visual symptoms that develop because of prolonged looking of a digital screen when the task's demands exceed the viewer’s capacities. The symptoms of CVS, also known as digital eye strain, include dry and irritated eyes, eye strain/fatigue, blurred vision, red eyes, burning eyes, excessive tears, double vision, headache, light/glare sensitivity, delay in shifting focus, and color perception irregularities [4].

Computer vision syndrome (CVS) is the most common 21st-century occupational hazard, which affects more than 70% of all computer users [5]. CVS is a serious public health issue that results in decreased workplace productivity, higher error rates, lower job satisfaction, and compromised visual ability. Approximately 60 million individuals globally suffer from CVS, with 1 million new instances occurring each year, according to statistics [6]. The problem of CVS is extremely high in underdeveloped nations because of the inadequate accessibility and use of equipment for personal protection, the high workload, and the restricted break time when using a computer [7].

Policymakers are increasingly concerned about the public health impact of CVS, and researchers are taking notice. According to research done in Abuja, Nigeria, 40% of computer users working as security and exchange commissioners had experienced at least one CVS symptom [8]. According to nationwide in Sri Lanka, more than two-thirds of computer office staff members have CVS [9]. According to a couple of surveys done in Gondar, Ethiopia, over 73 percent of computer users working as bankers, secretaries, and data processors were developing CVS [10, 11].

Although there is little confirmation that CVS symptoms cause lasting eye harm in addition to vision impairment, it does increase occupational inefficiencies. As a result, CVS is becoming an increasing public health concern that has the potential to negatively impact employees' quality of life and productivity [5].

Furthermore, the variables that contribute to computer vision syndrome among employees who use computers in Ethiopia and the symptoms of computer vision syndrome vary from one district to another. While there is little data in this area, the study outcomes are inconsistent; therefore, it is advisable to work toward evidence synthesis. This study aimed to estimate the overall magnitude of computer vision syndrome (CVS) among employees who use computers in Ethiopia and to identify risk factors for computer vision syndrome. This study provided light on the negative effects of computer usage, as well as preventative and control techniques, among computer users in Ethiopian government offices. The study could potentially be utilized as a foundation for investigators to conduct confirmatory inquiries. The review topic was: What are the cumulative magnitude and determinants of computer vision syndrome among employees who use computers in Ethiopia?

## Methods

### Protocol registration

This study’s protocol can be accessed via a web URL (https://www.crd.york.ac.uk/prospero/# my Prospero). In addition, the protocol identification number is CRD42022313924.

### Identification and study selection

Three researchers (FA, YM, and MD) searched both unpublished and published studies on the prevalence and predictors of computer vision syndrome among employees who use the computer in Ethiopia. The studies were identified using a search of Medline (Pub Med), HINARI, EMBASE, Google Scholar, Science Direct, Cochrane Library, and other databases. The reference list for each included article was also manually searched for search optimization. We had conducted the literature search from September 1, 2021, to November 30, 2021, and we have limited it to the English language. Google and Google Scholar were used to search for any unpublished papers. The search words were specified for a comprehensive search that covered all fields in records, as well as Medical Subject Headings (MeSH terms) to broaden the scope of the search in a PubMed advanced search. Within each axis, we combined keywords with the “OR” operator in the Boolean operator and then linked the two axes’ search techniques to the “AND” operator. “Prevalence” OR “Epidemiology” AND “Computer Vision Syndrome” OR “computer users” AND “Ethiopia” were the key phrases used in the search. The specific searching detail in PubMed with MeSH terms was (“int j Comput vis”[Journal] OR (“computer”[All Fields] AND “vision”[All Fields]) OR “computer vision”[All Fields]) AND (“syndrome”[All Fields] OR “syndromal”[All Fields] OR “syndromally”[All Fields] OR “syndrome”[MeSH Terms] OR “syndrome”[All Fields] OR “syndromes”[All Fields] OR “syndromes”[All Fields] OR “syndromic”[All Fields] OR “syndromes”[All Fields]) were used. The systematic review and meta-analysis include all types of literature available until November 30, 2021. The Preferred Reporting Items for Systematic Reviews and Meta-Analyses (PRISMA) criteria were used to conduct the systematic review and meta-analysis [12] (Table 1).

**Table 1:** Example of MEDLINE/PubMed and Google Scholar database searches to analyze the prevalence and predictors of Computer Vision Syndrome among employees who use the computers in Ethiopia

Sources	search engine	Number of studies
PubMed	MeSH terms was (“int j Comput vis”[Journal] OR (“computer”[All Fields] AND “vision”[All Fields]) OR “computer vision”[All Fields]) AND (“syndrome”[All Fields] OR “syndromal”[All Fields] OR “syndromally”[All Fields] OR “syndrome”[MeSH Terms] OR “syndrome”[All Fields] OR “syndromes”[All Fields] OR “syndromes”[All Fields] OR “syndromic”[All Fields] OR “syndroms”[All Fields])	63
Science Direct	((Computer vision syndrome OR vision disorders And predictors OR Associated factors) AND Ethiopia AND (incidence OR prevalence OR magnitude))	61
Google scholar	A combination of the above key terms (computer vision syndrome, vision disorder, eye problems, computer users, bank workers, secretaries, prevalence, magnitude, predictors, associated factor, Ethiopia)	118
Manual search		7
Research repositories		3
From other database		86
Total retrieved articles		338
Finally full articles relevant to our review		8

### Eligibility criteria

#### Inclusion criteria

Articles on the occurrence of computer vision syndrome and its predictors among employees who use computers in Ethiopia were considered.

*Study area* The articles were done in Ethiopia.

*Study design* All observational studies (cross-sectional, case–control, and cohort) containing original data on the prevalence and predictors of computer vision syndrome among employees who use computers in Ethiopia were examined.

*Language* Literature that was written in the English language.

*Population* Studies that have been considered among employees who use computers in Ethiopia.

*Publication condition* A consideration was given to both published articles and unpublished research.

*Exclusion criteria* Unpublished, internet-inaccessible studies were excluded. Also, we excluded studies whose corresponding authors did not respond to our email contact sent in quest of missing important data. Furthermore, after three writers (FA, YM, and MD) read the entire article, research that did not deliver our desired result was omitted.

### Data extraction

All essential data were extracted in Microsoft Excel TM using a checklist data extraction format made by three authors (FA, YM, and MD). The three authors’ independently extracted the data of each of the original articles using the checklist. The data extraction format for the prevalence of computer vision syndrome was produced based on the first author, the location where the study was conducted, the publication year, the sample size, and the prevalence of computer vision syndrome specified for the target population.

The data extraction format for predictors was customized for each predictor (previous history of eye disease, sitting position, using eyeglass/spectacles, and workload on the computer). The authors selected these variables, since they were the most frequently stated associated factors in the included articles in this study. Additional variables were included as risk factors in this systematic review and meta-analysis if they were explored as risk factors in two or more studies. Three researchers (FA, YM, and MD) gathered data from the primary studies in the form of two by two tables for each identified risk factor to compute the odds ratio.

### Outcome measurements

There are two major outcomes from this systematic review and meta-analysis. The primary outcome was the prevalence of computer vision syndrome among employees who use computers in Ethiopia. The secondary outcome of the study was the predictor of computer vision syndrome among workers using computers in Ethiopia. The prevalence was determined by dividing the number of participants with computer vision syndromes by the total number of computer users in the study (sample size) and multiplying the result by 100.

### Quality assessment

The Newcastle–Ottawa Scale adapted for cross-sectional study quality rating was used by the researchers (FA & YM) to assess the quality of the articles included in this study [13]. The tool is divided into three sections; the first, with five stars, evaluates the methodological excellence of each study. The second section of the instrument assesses study comparability and assigns two points. The final section, which can be rated out of three stars, assesses the original articles' consistency in terms of statistical analysis. The tool was used as a checklist to assess the quality of each of the primary articles. The two authors individually evaluated the quality of each of the original articles using the tool as a checklist. Any disagreements amongst the authors about the results of the quality evaluation were resolved through discussion. The quality of the articles in this study ranges from medium to high (6 out of 10 stars).

### Statistical analysis

The required data were mined in Microsoft Excel TM format and analyzed in STATA Version 15.0. The original studies were presented in the form of forest plots and tables. The authors used the binomial distribution method to calculate the standard error prevalence for each original article. The application of test heterogeneity *x*^2^, *I*^2^, and *p* values revealed heterogeneity among the recorded prevalence of studies [14]. The statistical analysis reported above found that the studies were significantly different (*I*^2^ = 68.4%, *p* value < 0.002). As a result, the combined effect of Der Simonian and Laird was estimated using a random effect meta-analysis approach. Moreover, a univariate meta-regression model was run using the year of publication and sample size to identify the likely source of heterogeneity, but none of the results was statistically significant. Egger’s correlation and Begg’s regression intercept tests were used to objectively screen for possible publication bias at a 5% significant level, respectively [13, 15]. Egger’s weighted regression and Begg’s rank correlation test methods were also used to assess publication bias (*p* > 0.05), revealing that publication bias was statistically insignificant. Furthermore, to minimize the random discrepancies between the primary study’s point estimations, subgroup analysis was undertaken based on the profession in which the studies were conducted.

## Results

### Search results

**Fig. 1:**
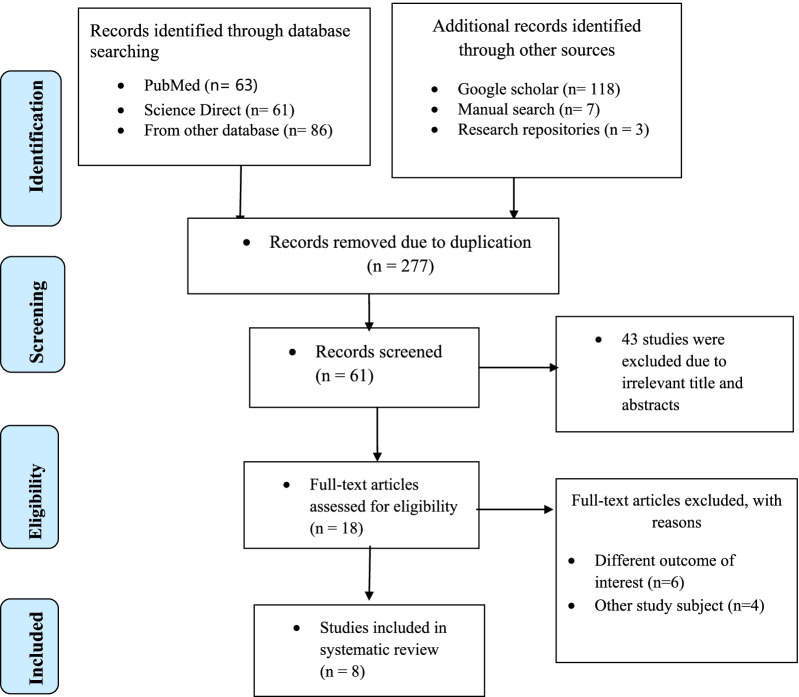
Flow diagram describing the selection of studies for the systematic review and meta-analysis of prevalence and associated factors of computer vision syndrome and associated factors among computer users in Ethiopia (showing how articles were identified, screened, and included in the studies), 2021

Searching the following databases yielded 338 publications on the prevalence and determinants of computer vision syndrome among employees who use the computer in Ethiopia: Medline (Pub Med), EMBASE, Science Direct, HINARI, Cochrane Library, Google Scholar, and other sources indicated above. Due to redundancy, 277 articles were eliminated from the preliminary records. After examining the titles and abstracts of the remaining 61 articles, 43 were judged non-applicable and were eliminated. The remaining 18 full-text papers were then obtained and assessed for eligibility using the preset criteria, resulting in the exclusion of 10 articles, mostly due to the research population and outcome of interest being ineligible. In addition to Ethiopia, four of these studies were conducted in other countries: Saudi Arabia [16], Ghana [17], Egypt [18], and Nepal [19]. The remaining six types of research were carried out in Ethiopia's various regions [20–25]. They were excluded due to the study population and the outcomes of interest were not measured or ineligible, because the results for the outcome of interest were not reported. The quality scores of each research review varied from 7 to 9 out of a possible 10 points; hence, no studies were excluded based on this criterion. Finally, the final meta-analysis included eight studies (Fig. 1).

### Characteristics of original articles

This study included eight original studies that revealed the prevalence and predictors of computer vision syndrome and determinant factors among employees who use computers in Ethiopia. The studies were carried out between 2014 and 2021. All of the included studies used cross-sectional designs. In this study, 2619 study participants were involved to assess the pooled prevalence of computer vision syndrome and determining factors among employees who use computers in Ethiopia.

The studies were conducted in the Amhara region [10, 26, 27], Oromia region[28], Addis Ababa [29–31], and Ethiopian university [31]. The sample sizes ranged from 128 from the study done at Ethiopian university [31] to 607 in another study conducted in the Amhara region [27] (Table 2).

**Table 2:** Descriptive summary of eight studies reporting the prevalence of computer vision syndrome and associated factors among employees who use the computers in Ethiopia included in the systematic review and meta-analysis

Author	Publication year	Region	Profession	Sample size	Case	Quality score (10 pts)	Prevalence with 95%
Lemma et al. [29]	2020	Addis Ababa	Secretaries	455	313	9	68.80 (64.54, 73.06)
Assefa et al. [26]	2017	Amhara	Bankers	304	222	9	73.00 (68.01, 77.99)
Dessie et al. [27]	2018	Amhara	office Employees	607	422	9	69.50 (65.84, 73.16)
Zenbaba et al. [32]	2021	Ethiopian universities	office Employees	128	27	9	70.40 (66.01, 74.79)
Alemayehu et al. [33]	2014	Amhara	Secretaries	284	210	9	73.90 (68.79, 79.01)
Tesfa et al. [28]	2021	Oromia	Secretaries	217	164	7	75.60 (69.89, 81.31)
Derbew et al. [30]	2021	Addis Ababa	Bankers	352	262	9	74.40 (69.84, 78.96)
Gondol et al. [34]	2018	Addis Ababa	Office employees	272	221	8	81.30 (76.67, 85.93)

### Meta-analysis

#### The prevalence of computer vision syndrome among employees who use computers in Ethiopia

**Fig. 2:**
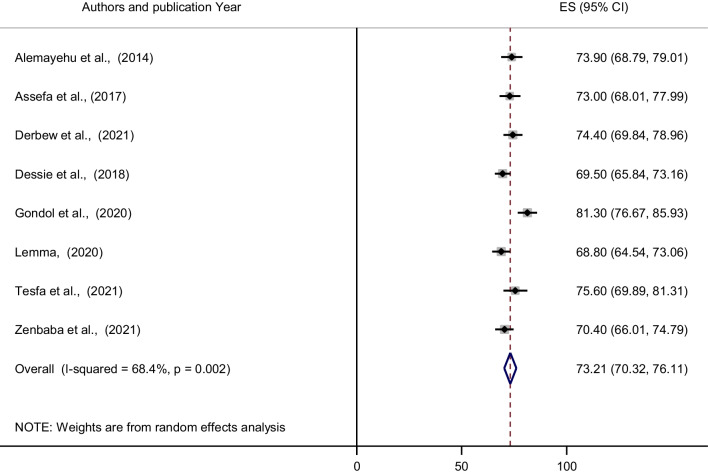
Forest plot of the pooled prevalence of computer vision syndrome among computer users in Ethiopia, 2021

Using eight studies conducted in Ethiopia, showed that the pooled prevalence of computer vision syndrome among employees who use the computers was 73.21% (95% CI 70.32–76.11) (Fig. 2). However, considerable heterogeneity was found across the studies as revealed by *I*^2^ statistic (*I*^2^ = 68.4%, *p* value < 0.002). To evaluate the pooled prevalence of computer vision syndrome among employees who use computers in Ethiopia, a random effect model was used. A univariate meta-regression model was also used to identify potential sources of heterogeneity by taking into account factors, such as publication year and sample size. None of these variables, however, was found to be statistically significant. According to Beggs and Eggers’ tests, there was no statistically significant publication bias (*p* > 0.05).

### Sub-group analysis

**Fig. 3:**
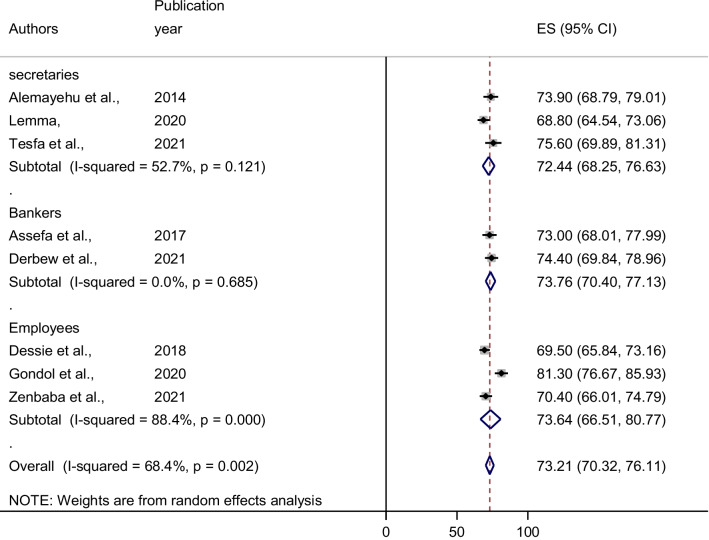
Forest plot of the Subgroup analysis of the prevalence of computer vision syndrome in different professions in Ethiopia, 2021

A profession-based sub-group analysis was performed to investigate the likely cause of heterogeneity among the studies due to significant heterogeneity among the articles included in this study. The sub-group analysis shows the highest prevalence was observed in bank employees with the prevalence of 73.76% (95% CI: 70.40–77.13) followed by office employees 73.64% (95% CI: 66.51–80.77), while the lowest prevalence was observed in secretaries 72.44% (95% CI 68.25–76.63) (Fig. 3).

#### The symptoms of computer vision syndrome (CVS) among employees who use computers in Ethiopia

**Fig. 4:**
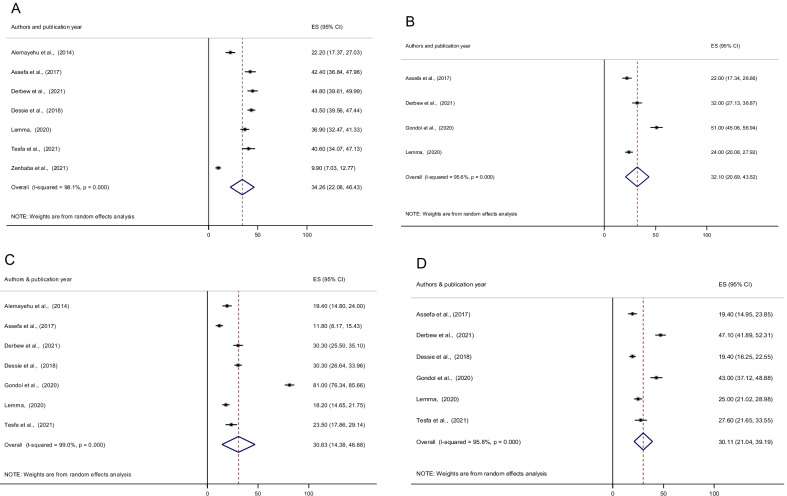

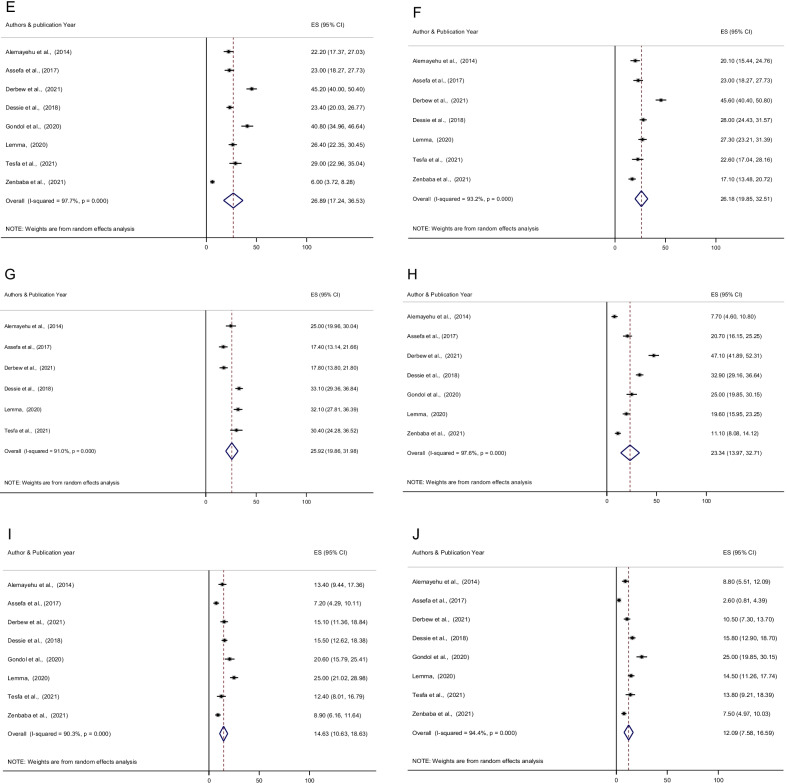
Forest plot depicting the pooled prevalence of symptoms of computer vision syndrome among computer users; (**A** Blurred vision, **B** Eyes Fatigue, **C** Watery eyes, **D** Burning sensation, **E** Headache, **F** Redness of the eyes, **G** Eyestrain, **H** Eye Irritation, **I** Dry eye, **J** Double vision) in Ethiopia, 2021

The prevalence of the most common reported symptoms of CVS were blurred vision (34.26%; 95% CI 22.08, 46.43) followed by eyes fatigue (32.10%; 95% CI 20.69, 43.52), watery eyes (30.63%; 95% CI 14.38, 46.88), burning sensation (30.11%; 95% CI 21.04, 39.19) and headache (26.89%; 95% CI 17.24, 36.53). In addition, redness of eyes (26.18%; 95% CI 19.85, 32.51), eyestrain (25.92%; 95% CI 19.86, 31.98), eyes irritation (23.34%; 95% CI 13.97, 32.71), dryness of eyes (14.63%; 95% CI 10.63, 18.63), and double vision (12.09%; 95% CI 7.58, 16.59) were also the symptoms of CVS (Fig. 4 and Table 3).

**Table 3:** Prevalence of computer vision syndrome symptoms among employees who use the computer in Ethiopia

No	Symptoms of computer vision syndrome	Prevalence (95% CI)
1	Blurred vision	34.26 (22.08, 46.43)
2	Eye fatigue	32.10 (20.69, 43.52)
3	Watery eyes	30.63 (14.38, 46.88)
4	Burning sensation	30.11 (21.04, 39.19)
5	Headache	26.89 (17.24, 36.53)
6	Redness of eyes	26.18 (19.85, 32.51)
7	Eye strain	25.92 (19.86, 31.98)
8	Eyes irritation	23.34 (13.97, 32.71)
9	Dry eyes	14.63 (10.63, 18.63)
10	Double vision	12.09 (7.58, 16.59)

### Predictors of CVS among computer users in Ethiopia

The previous history of eye disease (95% CI 2.30, 5.47), inappropriate sitting position (95% CI 1.76, 3.22), using computer more frequently (always/often) (95% CI 2.04, 3.60), and using eyeglass/spectacles (95% CI 1.10, 3.91) were significantly associated with computer vision syndrome among employees who use computer in Ethiopia.

**Fig. 5:**
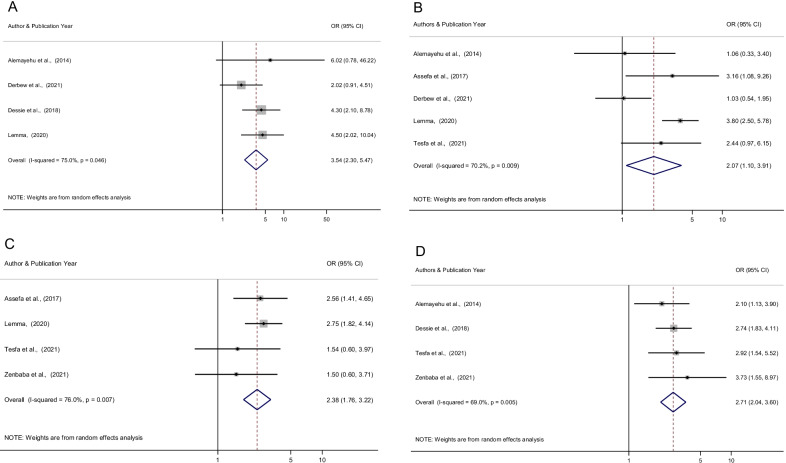
Forest plot depicting pooled odds ratio (log scale) of the associations between prevalence of computer vision syndrome and its predictors (**A** Previous history of eye disease, **B** Using eyeglass/spectacles, **C** Inappropriate sitting position, **D** Frequent use of the computer) in Ethiopia, 2021

In this study, the risk of computer vision syndrome was found to be 3.54 times higher among computer users with a history of eye disease than among computer users without a history of eye disease (OR = 3.54 [95% CI 2.30, 5.47]). In addition, computer users who had inappropriate sitting positions were 2.38 times more likely to have computer vision syndrome when compared with those who had appropriate sitting positions (OR = 2.38, [95% CI 1.76, 3.22]). Furthermore, those who used their computer more frequently (always/often) were 2.71 times more likely to develop computer vision syndrome than those who used their computer less frequently (rarely/occasionally) (OR = 2.71, [95% CI 2.04, 3.60]). Finally, the odds of having CVS among computer users who use eyeglass/ spectacles were 2.07 times more likely than those who did not use eyeglass (OR = 2.07, [95% CI 1.10, 3.91]) (Fig. 5A–J).

## Discussion

Computer vision syndrome (CVS) is a public health problem that is associated with the use of computers. In developing countries, such as Ethiopia, occupational health and safety, unfortunately, takes a back seat most of the time [35, 36]. This study aimed to determine the pooled prevalence of computer vision syndrome and predictors among employees who use computers in Ethiopia. According to the study, the overall prevalence of computer vision syndrome among employees who use computers in Ethiopia was 73.21%. This result is analogous to studies conducted in the United Arab Emirates [37] and Nigeria [8], which reported CVS magnitudes of 73.9% and 74%, respectively. On the other hand, the magnitude of CVS observed in the present study was lower than the prevalence reported (89%) in studies conducted in Malaysia [38] and the prevalence reported (80.3%) in Chennai [39]. Though the observed prevalence of CVS in this study was greater than the researches findings in Mauritius (59.3%) [40] and Malaysia (63%) [41]. This disparity could be explained by differences in study methods, study settings, data collection tools, study population, and computer safety awareness.

Sub-group analysis of this study revealed that the prevalence of computer vision syndrome among computer users varies across professions. Bank workers had the highest CVS prevalence, followed by office employees, and secretaries had the lowest. The reason for this could be that bank employees use computers and other electronic devices for longer periods than office and secretary employees do[28, 42].

In our study, the most commonly reported symptoms of CVS were blurred vision, eye fatigue, watery eyes, a burning sensation, and a headache. The previous research in India [43], Pakistan [44], Brazil [45], and Nigeria [8] found that computer users are at a high risk of developing visual symptoms. Furthermore, according to a study conducted in the United Arab Emirates [37], the most commonly reported symptoms were burning sensation, headache, and dry/sore/tired eyes.

Furthermore, the current study reveals that there are significant associations between computer vision syndrome and previous history of eye disease, inappropriate sitting position, computer workload (doing work by a computer usually/always), and use of eyeglasses/spectacles. In this study, the likelihood of computer vision syndrome was approximately 3.54 times higher among computer users who had a previous history of eye disease than those who had no history of eye disease. This outcome was consistent with findings from previous studies in Sri Lanka and India [9, 46]. This could be a long-term side effect of a previous disease; the illness could have been persistent until now, producing CVS, a lack of care, and therapy for the previous illness, and some of the previous difficulties could have been chronic and present until now.

The current study found that the inappropriate sitting position of computer users was 2.38 times more likely to be associated with CVS than an appropriate sitting position during the use of computers. This could be explained by the fact that an incorrect seating position produces ocular discomfort and tension, causing the eye to become more concentrated and the eye muscles to spasm, resulting in CVS symptoms. Computer users, on the other hand, identify glare as their primary issue while using a computer when they are seated incorrectly. It is also possible to justify that eye and environment-related conditions arise as a result of an incorrect sitting position, when the task’s viewing demands exceed the user’s visual abilities, resulting in an inability to focus properly on computer images and experience CVS [5].

In the present study, computer vision syndrome was 2.71 times more likely in those who used their computer more frequently (always/often) than those who used it less frequently (rarely/occasionally). The current finding is consistent with previous research, which suggests that the amount of hours consumed on a computer rises the risk of CVS [9, 47, 48]. As a result, reducing the amount of time spent on the computer is critical for preventing CVS [49]. This may be because a computer generates electromagnetic radiation or high-energy blue light, which stresses the ciliary muscle in the eye, resulting in eye strain after prolonged exposure to the computer screen [49].

Finally, CVS was 2.07 times more likely in computer users who wore eyeglasses/ spectacles than in those who did not. Similar findings were reported in Malaysia [38], where computer users who wore spectacles experienced CVS significantly more frequently than those who did not do. The higher prevalence of CVS among eyeglass wearers could be explained by the fact that spectacles were not prescribed by professionals, resulting in either incorrect prescription or the lack of glare or reflection protection surfaces.

As a result, raising the knowlege of the community about the growing prevalence of computer vision syndrome and its symptoms is crucial for preventing the emergence of computer vision syndrome when using a computer. To avoid the occurrence of CVS, computer users should be encouraged to take frequent breaks, correct their posture, and use ergonomics. The key activities in preventing CVS are educating the public about the dangers of wearing eyeglasses/spectacles without a physician prescription for computer users and warning those with eye problems not to use a computer until their condition is cured.

### Strengths and limitations of the study

The strength of this study is that it is the first of its kind in Ethiopia, and it is based on a search for existing and unpublished research, as well as the utilization of diverse perspectives to strengthen the study.

The studies in this systematic review and meta-analysis, on the other hand, are all cross-sectional. As a result, establishing temporal correlations between causes and outcome variables is impossible. The majority of the research included in this evaluation had small sample sizes, which could affect the final estimate. Furthermore, since this meta-analysis contained research from a small portion of Ethiopia, the country's many regions may have been under-represented. Some regions, including Harari, Afar, Benshangul Gumze, Dire-Dawa, and Somalia, have no data. As a result, the results might not be representative of the aforementioned areas. Another limitation could be the possibility of missing research, because not all databases were accessible.

## Conclusions

According to this study, the prevalence of CVS among computer users was found to be higher in Ethiopia. Bank employees had the highest CVS prevalence, followed by office employees and secretaries. Blurred vision, eye fatigue, watery eyes, a burning sensation, and a headache were the most commonly experienced visual-related problems.

The previous history of eye disease, inappropriate sitting position, frequently use of a computer, and use of eyeglasses/spectacles were significantly associated with CVS among employees who use computers. Based on the findings, it is recommended that efforts should be made to optimize computer exposure time. It is also important to note that when using a computer, employees should be properly seated. It is also recommended that people take caution while acquiring eyeglasses/spectacles without a physician’s prescription. Furthermore, it is critical to raise community awareness of the safety precautions that can be taken to reduce CVS. Finally, when using a computer, people with vision problems should be extra cautious.

## Data Availability

The data sets used and/or analyzed during the current study are available from the corresponding author on reasonable request.

## Abbreviations

CVS: Computer vision syndrome
AOA: American optometric association
MeSH: Medical subject headings
PRISMA: Preferred Reporting Items for Systematic reviews and Meta-Analyses
